# Automated Lewis Score Estimation for Small Bowel Crohn Disease Using Capsule Endoscopy: Retrospective Cohort Study

**DOI:** 10.2196/92416

**Published:** 2026-10-13

**Authors:** Yingying Meng, Min Gao, Jie Wu, Ying Huang

**Affiliations:** 1 Children's Hospital of Fudan University Shanghai China; 2 Chongqing Jinshan Technology (Group) Co, Ltd Chongqing China

**Keywords:** Crohn disease, small bowel capsule endoscopy, Lewis score, deep learning, endoscopic image analysis, automatic scoring, lesion segmentation, medical artificial intelligence, artificial intelligence, AI

## Abstract

**Background:**

Small bowel capsule endoscopy (SBCE) is widely used to evaluate small bowel involvement in Crohn disease (CD); however, manual Lewis score calculation is time-consuming and susceptible to interobserver variability.

**Objective:**

This study aimed to develop and preliminarily validate a multitask deep learning system for automated Lewis score estimation in patients with small bowel CD undergoing SBCE.

**Methods:**

We developed Eval-Model, a multitask framework integrating You Only Look Once (YOLO)–style lesion detection and localization for ulcers and edema, a stenosis classification branch, and a long short-term memory–based temporal regression module. The primary dataset comprised SBCE examinations from 814 patients at the Children’s Hospital of Fudan University. In this dataset, 725 (89.1%) cases were included for lesion-level annotation, and 89 (10.9%) cases with expert-consensus Lewis scores were used for scoring model development and internal testing. These 89 cases comprised 49 (55.1%) training cases and 40 (44.9%) internal test cases. All splits were performed at the patient level. A retrospective same-center temporal validation cohort of 20 consecutive cases from 2026 and 5 control cases were used for preliminary assessments of temporal robustness and specificity.

**Results:**

In the 40-case internal Lewis score test set, Eval-Model achieved a mean squared error of 14.693 (95% CI 8.72-22.15), root mean squared error of 3.833 (95% CI 2.95-4.71), mean absolute error of 2.9 (95% CI 2.1-3.8), *R*^2^ of 0.82 (95% CI 0.70-0.90), and explained variance of 0.83 (95% CI 0.72-0.91). For lesion recognition, the model achieved an overall mean average precision of 0.94 with an inference time of 15 ms per frame. In the 20-case same-center temporal validation cohort, the mean squared error was 26.848 (95% CI 12.51-45.33), the root mean squared error was 5.182 (95% CI 3.54-6.73), the mean absolute error was 3.3 (95% CI 2.2-4.6), *R*^2^ rounded to 1.00 (95% CI 0.98-1.00), and the explained variance rounded to 1.00 (95% CI 0.98-1.00). These temporal estimates require cautious interpretation because of the small, same-center cohort.

**Conclusions:**

Eval-Model demonstrated preliminary feasibility for automated Lewis score estimation from SBCE in small bowel CD. The system may serve as a standardized adjunct to expert review, but larger multicenter prospective validation with independent external annotation is required before clinical deployment.

## Introduction

Crohn disease (CD) is a chronic transmural granulomatous inflammatory disease that can affect the entire digestive tract. Approximately 70% of CD cases involve the small intestine, and nearly 30% of lesions are confined solely to the small intestine [1,2]. Assessment of small bowel inflammation is clinically important because mucosal activity influences treatment selection, monitoring strategies, and prognosis.

Small bowel capsule endoscopy (SBCE) effectively observes CD lesions in the small intestine and determines the extent of the lesions. However, a single SBCE examination commonly generates tens of thousands of frames, making manual review time-consuming and dependent on reader experience [3]. Image quality can also be affected by bile, bubbles, food residue, and incomplete mucosal visualization, which may further contribute to variability in lesion interpretation.

The Lewis score (LS) is widely used to quantify inflammatory activity in small bowel CD based on villous edema, ulceration, and stenosis [4]. Although clinically useful, manual LS calculation requires careful review of long SBCE sequences and is susceptible to interobserver variability. Automated estimation of LS may therefore improve consistency and reduce the workload associated with SBCE interpretation.

Deep learning has demonstrated substantial promise for automated capsule endoscopy analysis, particularly in the detection of ulcers, bleeding, polyps, vascular lesions, and other mucosal abnormalities [5,6]. Beyond diagnostic detection, deep learning algorithms can characterize inflammatory features on endoscopic images to quantify lesion severity, whereas machine learning models can predict the risk of disease recurrence [7,8]. Most existing models, however, focus on single-frame or single-task lesion recognition and do not directly integrate lesion detection, stenosis assessment, and the sequence-level temporal information required for LS estimation.

To address this gap, we developed and preliminarily validated Eval-Model, a multitask deep learning framework integrating lesion detection and localization, stenosis classification, and long short-term memory (LSTM)–based temporal regression for automated LS estimation in SBCE examinations of small bowel CD.

## Methods

### Model Architecture

Eval-Model (Figure 1) was designed as a multitask deep learning framework inspired by You Only Look Once (YOLO)–style object detection networks [9] and recurrent neural networks [10]. We introduce a study-specific detection backbone named YOLO-v11 (a customized variant, not the official version), which features a modified cross-stage partial (CSP) backbone with C3K2 blocks incorporating sigmoid linear unit (Swish) activation, a spatial pyramid pooling–fast (SPPF) and spatial attention–augmented neck, and a 3-scale head (80 × 80, 40 × 40, and 20 × 20) for size-variable lesion localization using 256 × 256 inputs and complete intersection over union (CIoU) loss. The CSP design divides feature maps along the channel dimension into separate paths that are subsequently merged to reduce redundant gradient information. SPPF applies repeated max pooling with a fixed kernel size and concatenates the input and pooled feature maps to aggregate information across multiple receptive fields. CIoU combines bounding box overlap with penalties for normalized center-point distance and aspect-ratio mismatch between predicted and ground-truth boxes. On top of this backbone, Eval-Model branches into a segmentation head for pixelwise edema and ulcer identification and a classification head for stenosis. The features from both heads are then concatenated and fed into an LSTM module to capture temporal dependencies across sequential capsule endoscopy frames, ultimately producing a predicted examination-level LS that reflects both spatial lesion patterns and their evolution over the image sequence. Thus, the model unifies detection, segmentation, classification, and sequential scoring within a single end-to-end framework.

**Figure 1:**
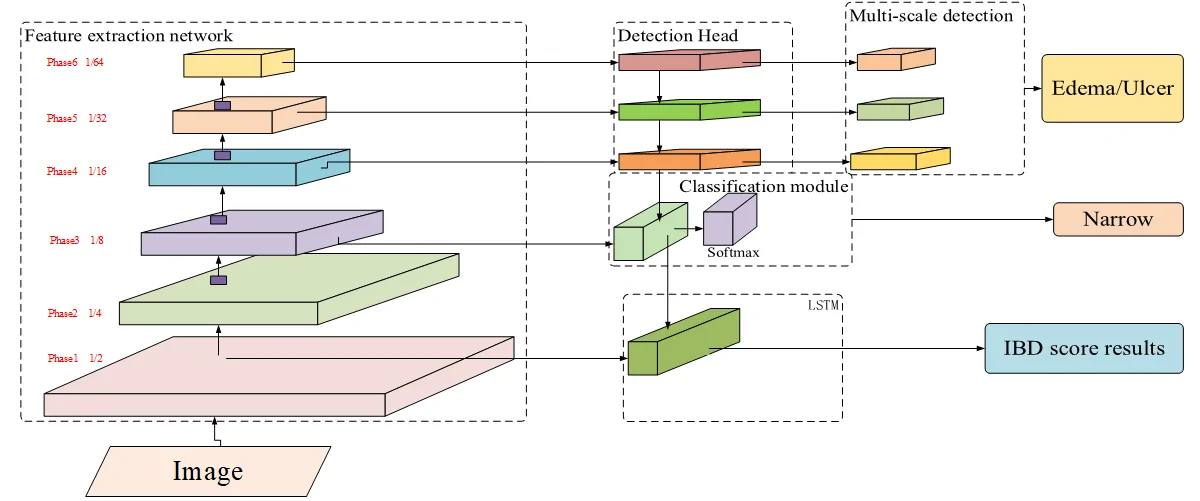
Architecture of the Eval-Model network framework. Phase 1 to phase 6 represent feature map levels at 1/2, 1/4, 1/8, 1/16, 1/32, and 1/64 of the input image resolution, respectively. The outputs are edema and ulcer detection (edema and/or ulcer), stenosis classification (narrow), and Lewis score estimation (IBD score results). IBD: inflammatory bowel disease; LSTM: long short-term memory.

The mathematical definition of the sigmoid linear unit activation function is provided in equation 1:

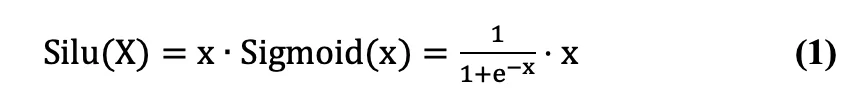



The lesion detection and localization module comprises 3 detection heads that operate on top-down, multiscale features extracted by the backbone network. These features are further processed through a bottom-up path aggregation network to generate lesion localization outputs at multiple scales.

The multiscale detection features are concatenated and passed to a stenosis classification branch consisting of 10 convolutional units. Each unit includes a convolutional layer, a pooling layer, and layer normalization, enabling image-level stenosis classification.

The scoring module is an LSTM-based temporal regression component. It integrates lesion detection features and stenosis-related features across the SBCE sequence and outputs a single continuous value representing the predicted LS. Batch normalization and rectified linear unit activation are applied after the LSTM convolutional layers.

During training, the learning rate followed a polynomial decay schedule defined as initial learning rate × (1 – iter/max_iter_size)^power, with an initial learning rate of 0.03 and power of 0.5.

The LS was modeled according to its original clinical components: villous edema, ulceration, and stenosis. Edema is scored according to the number and extent of affected villi; ulceration is scored according to the number, size, and depth of ulcers; and stenosis is scored according to the presence and severity of luminal narrowing. The total score is derived from segment-level scores according to the original LS formula [4]. Eval-Model was therefore trained to reproduce the clinical LS rather than define a new scoring system.

### Temporal Sequence Construction and Aggregation Strategy

Each SBCE sequence was resampled at 1 frame per second (fps), half the device’s lowest effective acquisition rate of 2 fps, to reduce adjacent-frame redundancy. At an average small bowel transit time of approximately 3.5 hours, this produced approximately 12,600 time-ordered frames before quality filtering. The sampled small bowel sequence was divided by elapsed time into 3 equal-duration tertiles (proximal, middle, and distal). From each tertile, 300 contiguous frames were selected around its temporal midpoint. During training, the center of this window was randomly jittered by up to 50 frames in either direction; during inference, the fixed central window was used. Tertiles with fewer than 300 available frames were zero padded. The 3 windows were concatenated in proximal-to-distal order to form a fixed 900-frame sequence, and learnable positional encodings were added to the feature vectors to identify tertile membership.

For each sampled frame, the lesion detection and segmentation and stenosis branches generated frame-level feature vectors. The LSTM processed the concatenated 900-frame sequence in temporal order, and its final hidden state was passed to a fully connected regression head to generate one examination-level LS. Separate tertile scores were not calculated or averaged; aggregation occurred through the LSTM state after all 3 tertile windows had been processed. The 900 sampled frames represented approximately 7% (900/12,600, 7.1%) of a typical 3.5-hour small bowel recording at 1 fps. Thus, the strategy preserved broad proximal-to-distal distribution while remaining computationally feasible, but it did not provide exhaustive frame-level coverage.

### Research Dataset

The Eval-Model was developed using a self-built private SBCE dataset collected from 814 patients who underwent capsule endoscopy at the Children’s Hospital of Fudan University between January 2014 and October 2025. The OMOM Capsule (model SC100; Chongqing Jinshan Science & Technology Group) was set to use adaptive intelligent frequency, with a maximum frame rate of 8 fps and a minimum frame rate of 2 fps, an operating time exceeding 12 hours, and a 160 ° field of view. After data cleaning, 201,860 SBCE images with a resolution of 512 × 512 pixels were retained. All patient data were anonymized and deidentified.

Data annotation was performed by 3 experienced SBCE readers (YM, JW, and YH), with a mean SBCE reading experience of 10.0 (SD 5.3) years, and a mean annual SBCE review volume of 502.3 (SD 383.6) cases. For lesion recognition annotation, representative lesion frames were selected; ulcer and edema regions were localized; stenosis was classified; and frames with severe bubbles, residues, or obstruction were excluded. For the LS subset, expert-consensus scores were used as the reference standard. Interobserver reliability for LS annotation was assessed using the intraclass correlation coefficient, which was 0.91 (95% CI 0.85-0.95), indicating excellent agreement before consensus resolution.

The annotated data were organized at 4 levels: patient, video and frame, lesion annotation, and LS. The lesion dataset (Table 1) included 725 patient cases for ulcer, edema, and stenosis recognition. The LS dataset (Table 2) included 89 patient cases, of which 49 (55.1%) were used for scoring model training and 40 (44.9%) were used for internal testing. All training, validation, and testing splits were performed at the patient level to minimize data leakage from adjacent frames or repeated images from the same patient.

**Table 1:** Composition of the lesion dataset.

	Ulcers, n (%)	Edema, n (%)	Stenosis, n (%)
Training set (n=507)	201 (39.6)	276 (54.4)	30 (5.9)
Testing set (n=218)	101 (46.3)	101 (46.3)	16 (7.3)

**Table 2:** Composition of the Lewis score dataset (n=89).

Dataset type	Cases, n (%)
Training set	49 (55.1)
Testing set	40 (44.9)

### Data Preprocessing

Images were preprocessed before model training. First, images were resized to 256 × 256 × 3 to reduce computational burden and facilitate efficient inference. Second, geometric data augmentation, including rotation and tilt transformation, was applied to increase image diversity and reduce overfitting. Third, pixel values were normalized to improve optimization stability and accelerate model convergence.

During model training, the training data were dynamically divided into training and validation subsets at a ratio of 70:30 to monitor optimization and generalization performance.

### Experimental Setup

The model was implemented in Python (version 3; Python Software Foundation) using the PyTorch framework (Meta AI). All images were preprocessed through a PyTorch image data pipeline, including augmentation, resizing, and normalization. Feature extraction weights were initialized using weights pretrained on the COCO object detection dataset. The key implementation details are reported above, including input resolution, feature extraction components, output scales, and bounding box regression loss.

Training was performed in 2 stages. In the first stage, lesion recognition components for edema, ulcers, and stenosis were trained for 100 epochs. In the second stage, the lesion recognition modules were frozen, and the scoring module was trained for 50 epochs.

Model optimization used stochastic gradient descent with a weight decay of 0.0001 and momentum of 0.9. Each model was trained for approximately 600 epochs, with 8 iterations per epoch and a batch size of 8. The trajectories of loss, precision, recall, and mean average precision (mAP) during training are shown in Figure 2.

**Figure 2:**
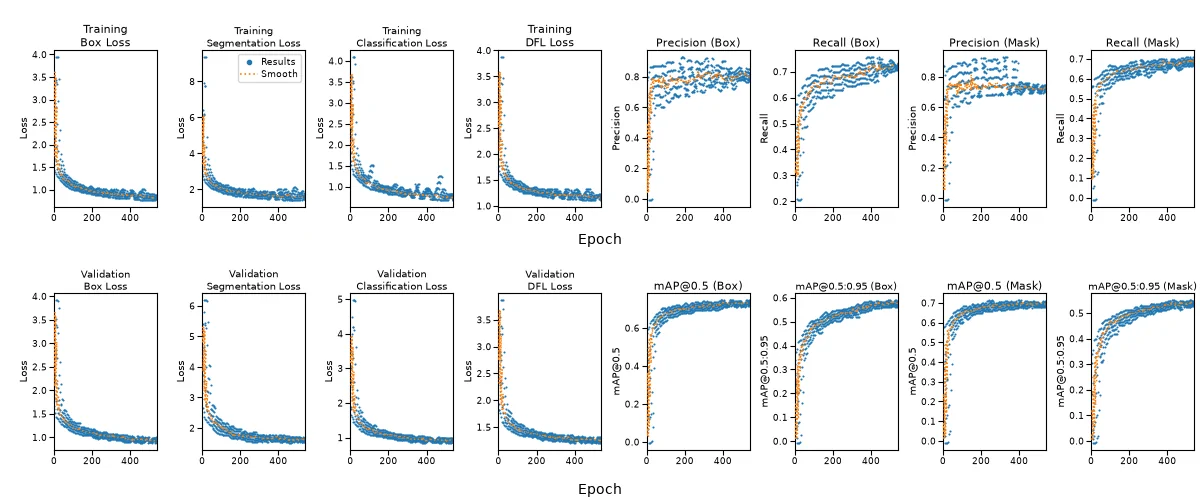
Training and validation losses and box and mask performance metrics across training epochs. DFL: distribution focal loss; mAP: mean average precision.

During training, loss decreased progressively in both the training and validation sets, whereas performance metrics improved with increasing training iterations. These trends suggested stable optimization without obvious underfitting or overfitting.

Loss denotes the optimization objective, precision and recall were monitored for box and mask predictions, and mAP was monitored for these predictions. The lesion recognition performance of Eval-Model on representative test set images is illustrated in Figure 3. Representative examples demonstrate that the Eval-Model can localize ulcerative lesions in SBCE frames.

**Figure 3:**
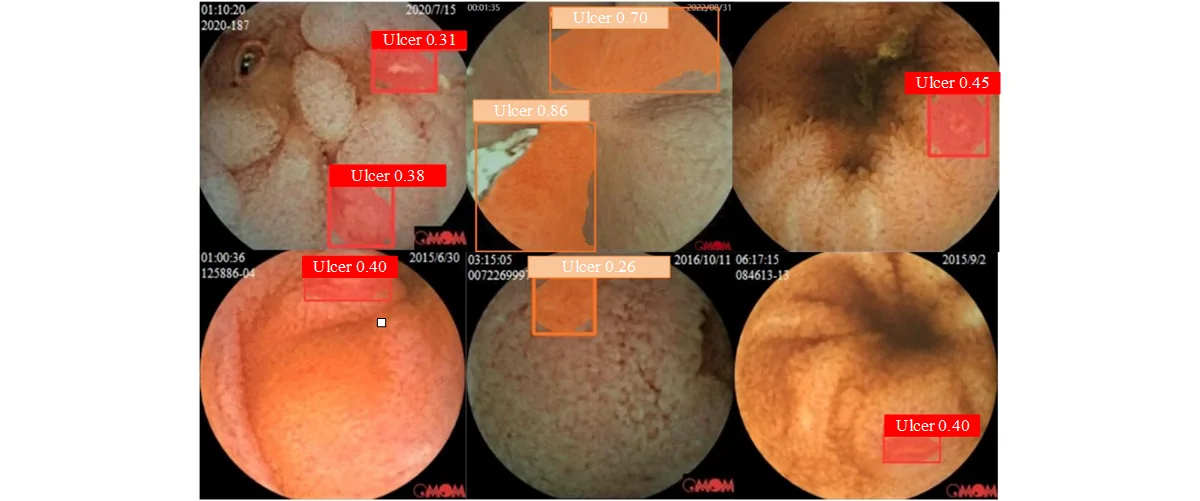
Representative ulcer detection results of the multitask recognition model.

### Evaluation Metrics

Eval-Model was evaluated on 2 major tasks: lesion detection and localization and LS regression. Lesion recognition performance was assessed using mAP. LS prediction was evaluated using mean squared error (MSE), root mean squared error (RMSE), mean absolute error (MAE), *R*^2^, and explained variance. MSE was calculated as shown in equation 2. To quantify uncertainty, 95% CIs for all score regression metrics were estimated separately for the 40-case internal test cohort and the 20-case same-center temporal validation cohort using 1000 nonparametric patient-level bootstrap resamples drawn with replacement. The patient examination was the resampling unit, and the 2.5th and 97.5th percentiles of the bootstrap distributions defined the interval limits.

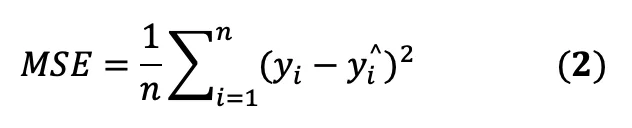



In this equation, *y_i_* denotes the expert reference LS, *ŷ_i_* denotes the model-predicted LS, and *n* denotes the number of evaluated cases.

Model complexity and deployment feasibility were described using parameter count, floating point computational complexity, and inference time.

### Ethical Considerations

This study was conducted in accordance with the ethical guidelines of the Declaration of Helsinki and was approved by the ethics committee of the Children’s Hospital of Fudan University (approval 2022; clinical ethics review No. 415). Informed consent was obtained.

## Results

The lesion recognition performance of Eval-Model was compared with that of 4 currently high-performing object detection models, as shown in Table 3.

**Table 3:** Comparison of lesion recognition performance among models^a^.

Network type	mAP^b^	Time used (ms)
Fast R-CNN^c^ [11]	0.72	185
SPPNet [12]	0.80	121
YOLO^d^-v5	0.81	15
YOLO-v7	0.89	16
Eval-Model	0.94	15

^a^mAP values are averaged across the ulcer, edema, and stenosis categories; inference time is reported in milliseconds per frame.

^b^mAP: mean average precision.

^c^R-CNN: region-based convolutional neural network.

^d^YOLO: You Only Look Once.

Eval-Model achieved the highest mean average precision among the compared models while maintaining an inference time of 15 ms. However, deployment feasibility should be interpreted together with parameter count and computational complexity.

Table 4 compares model parameter count and floating point computational complexity across the detection models and Eval-Model.

**Table 4:** Model parameter count and computational complexity.

Network type and network			Parameter count (in millions), n		Float computational complexity (in billions)
**Detection network**					
	Fast R-CNN^a^	179		987.23	
	SPPNet	168		899.43	
	YOLO^b^-v5	176.4		145.66	
	YOLO-v7	158.9		132.44	
Eval-Model			143.8		411.8

^a^R-CNN: region-based convolutional neural network.

^b^YOLO: You Only Look Once.

Category-specific recall and precision values for Eval-Model are summarized in Table 5.

**Table 5:** Lesion detection metrics by category.

Lesion category	Recall (%)	Precision (%)
Ulcer	76.7	93.8
Edema	82.1	99.2
Stenosis	95.7	98.8

To examine the contribution of individual components, an ablation analysis was performed by removing key modules and evaluating each resulting model on the same 40-case LS test set. The results are shown in Table 6.

**Table 6:** Ablation study results.

Configuration	MSE^a^	*R* ^2^
Eval-Model	14.69	0.82
Without YOLO^b^-v11 detector	28.41	0.61
Without LSTM^c^ temporal module	22.37	0.68
Without classification head	18.53	0.75

^a^MSE: mean squared error.

^b^YOLO: You Only Look Once.

^c^LSTM: long short-term memory.

Ablation analysis showed that each module contributed to scoring performance. Removing the YOLO-v11 detector reduced *R*^2^ from 0.82 to 0.61, indicating the importance of spatial lesion features. Removing the LSTM temporal module reduced *R*^2^ to 0.68, supporting the value of sequence-level lesion distribution modeling. Removing the stenosis classification branch reduced *R*^2^ to 0.75, suggesting that stenosis information also contributed to LS estimation.

The performance of Eval-Model on the 40-case internal LS test set is shown in Figure 4. The model achieved an MSE of 14.693 (95% CI 8.72-22.15), RMSE of 3.833 (95% CI 2.95-4.71), MAE of 2.9 (95% CI 2.1-3.8), *R*^2^ of 0.82 (95% CI 0.70-0.90), and explained variance of 0.83 (95% CI 0.72-0.91). These metrics should be interpreted as measures of agreement with expert reference scores rather than evidence of superiority over expert assessment. The CIs quantify resampling uncertainty within this small internal cohort and do not address transportability to other centers or devices.

**Figure 4:**
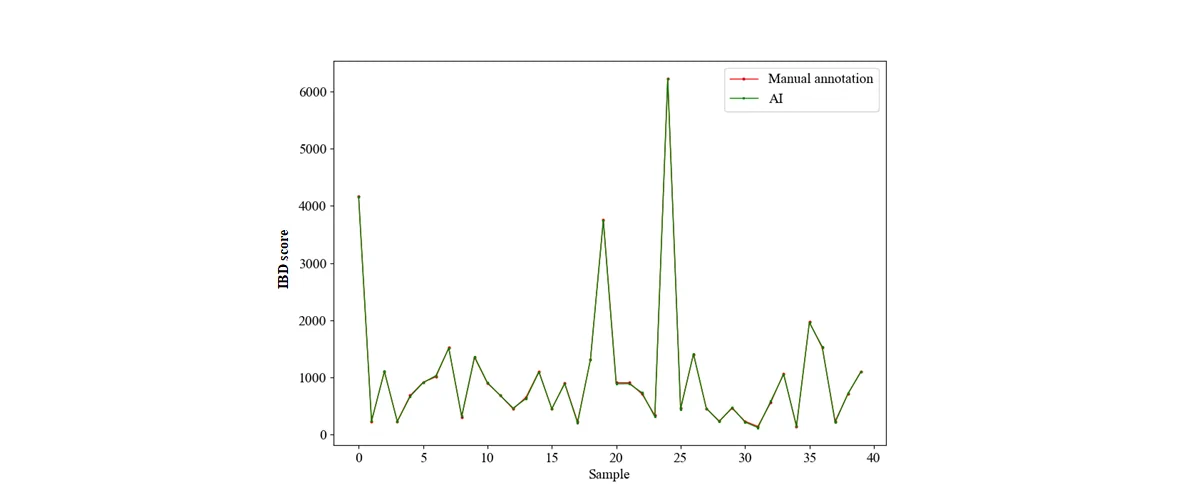
Agreement between automated and expert Lewis scores in the internal test set. Manual annotation denotes expert reference scores; AI denotes Eval-Model predictions. IBD: inflammatory bowel disease.

To enhance interpretability, Gradient-Weighted Class Activation Mapping (Grad-CAM) was used to visualize image regions contributing to LS prediction. The representative heat maps in Figure 5 show that the model primarily attended to ulcerated mucosa, edematous villi, and luminal narrowing, with attention patterns broadly corresponding to clinically relevant lesion regions identified by expert readers.

**Figure 5:**
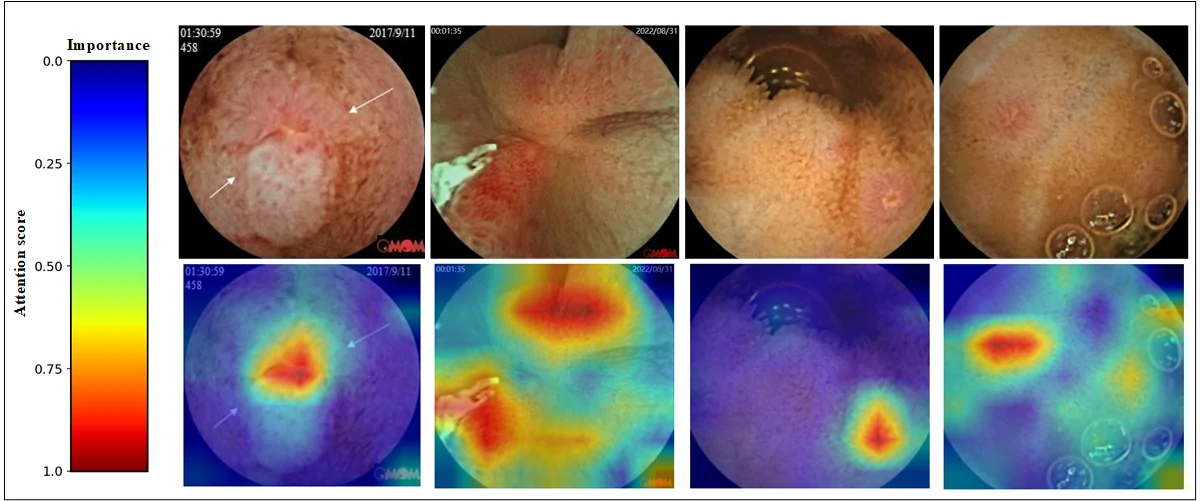
Gradient-Weighted Class Activation Mapping heat maps for model interpretability.

To preliminarily assess temporal robustness beyond the development and internal testing cohorts, 20 consecutive cases from 2026 were retrospectively collected as an independent same-center temporal validation cohort. These cases were obtained from the same institution and processed using the same capsule endoscopy system and annotation protocol, with expert LSs serving as the reference standard. Accordingly, this analysis should not be interpreted as multicenter external validation. The model was applied to this cohort without retraining or fine-tuning.

In the same-center temporal validation cohort, Eval-Model achieved an MSE of 26.848 (95% CI 12.51-45.33), RMSE of 5.182 (95% CI 3.54-6.73), MAE of 3.3 (95% CI 2.2-4.6), *R*^2^ rounded to 1.00 (95% CI 0.98-1.00), and explained variance rounded to 1.00 (95% CI 0.98-1.00). The higher MSE than in the internal test set indicates larger absolute errors in some cases. Although the variance-based metrics were close to 1.00, they were estimated from only 20 same-center cases and should not be interpreted as evidence of perfect prediction or external generalizability. The CIs quantify uncertainty within the observed cohort but do not remove the limitations of its small size and same-center design. The agreement between automated and expert LS in this cohort is shown in Figure 6.

**Figure 6:**
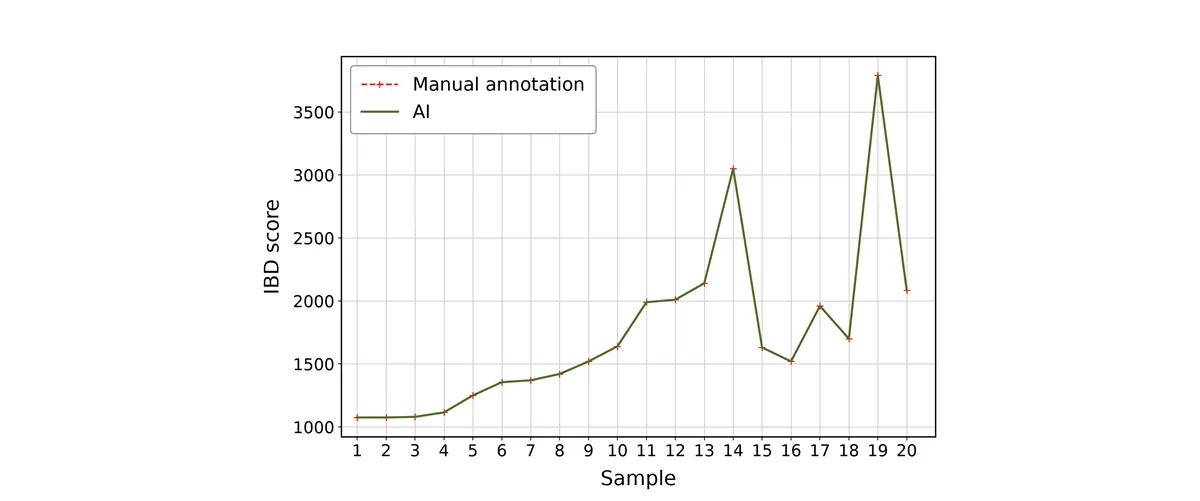
Automated and expert Lewis scores in the same-center temporal validation cohort. Manual annotation denotes expert reference scores; AI denotes Eval-Model predictions. IBD: inflammatory bowel disease.

These same-center temporal validation results are preliminary. Prospective multicenter validation involving heterogeneous patient populations, different capsule endoscopy systems, and independent external annotators is required to establish model robustness and generalizability.

To preliminarily assess specificity, 5 control cases without endoscopic evidence of CD-related inflammatory lesions were evaluated. Eval-Model produced automated scores of 1, 3, 3, 1, and 0, with a mean of 1.6 (SD 1.34). Among these 5 cases, 4 (80%) received nonzero scores, indicating low-level false-positive predictions in this preliminary analysis. The results are shown in Table 7.

**Table 7:** Control experiment results.

ID	Expert Lewis score	Automated Lewis score
1	0	1
2	0	3
3	0	3
4	0	1
5	0	0

These false-positive results may be explained by physiological mucosal variation, image artifacts such as bubbles or bile, sensitivity of the regression module to minor spatial feature deviations, and the limited representation of control cases in the training dataset.

Importantly, all automated scores in these control cases were low (≤3) and far below the clinical threshold for mild disease (LS≥135). Thus, although the model may generate low-level false-positive scores, it did not produce clinically misleading high scores in this preliminary control analysis. Future training should incorporate such control cases and confidence thresholds to improve specificity and support manual review of equivocal cases.

## Discussion

### Principal Findings and Methodological Contributions

This study developed and preliminarily validated Eval-Model, a multitask deep learning framework for SBCE-based lesion detection and localization and automated LS estimation in small bowel CD. In the internal test set, the model achieved recall values exceeding 75% and precision values exceeding 93% for villous edema, ulcers, and stenosis. For LS estimation, the model achieved an MSE of 14.693 and an *R*^2^ of 0.82. These results should be interpreted as evidence of preliminary agreement with expert reference scores rather than superiority over expert assessment.

The principal methodological contribution of this study is the integration of lesion detection and localization, stenosis classification, and sequence-level LS regression within a single framework. By sharing the YOLO-style feature extractor and incorporating LSTM-based temporal modeling, the model reduces the need to stack multiple independent task-specific models. Although Eval-Model had fewer parameters than the compared fast region-based convolutional neural network, SPPNet, YOLO-v5, and YOLO-v7 models, its computational complexity remained higher than that of YOLO-v5 and YOLO-v7; therefore, lightweight performance should be interpreted primarily in terms of parameter count rather than overall computational burden.

The LSTM module allowed the model to incorporate lesion distribution across SBCE sequences rather than relying solely on isolated frames. This design is clinically relevant because the LS reflects segment-level lesion burden and stenosis patterns across the small bowel, not merely the presence of individual lesions in selected images.

The scoring dataset of 89 patient cases enabled preliminary development and internal testing of automated LS estimation. However, the limited sample size and same-center design mean that the current results should be considered hypothesis generating and require external confirmation.

### Clinical and Practical Implications

SBCE is important for evaluating small bowel lesions in CD but requires substantial physician time and expertise. Automated LS estimation may improve assessment consistency, support less experienced readers, and facilitate longitudinal monitoring in clinical practice or trials. At the current stage, Eval-Model should be regarded as a decision support tool intended to assist rather than replace physician interpretation.

Previous convolutional neural network models have demonstrated robust performance in detecting small bowel lesions, including inflammation, bleeding, vascular lesions, ulcers, and stenosis [13-15]. AI-assisted interpretation has been shown to significantly improve the overall diagnostic accuracy of general endoscopists, achieving performance comparable to that of expert endoscopists [6,16] and identifying lesions missed during manual capsule endoscopy review [17]. However, most existing AI systems are limited to single-frame or single-lesion analysis tasks. In contrast, the proposed Eval-Model integrates lesion detection and localization with temporal aggregation and LS estimation, thereby more closely recapitulating the clinical scoring workflow used in SBCE assessment.

The LS incorporates quantitative parameters such as ulcers, villous edema, and stenosis, enabling a good assessment of small bowel CD activity and prediction of disease recurrence [18-20]. Several studies have explored the use of AI for CD severity assessment. Barash et al [21] developed a model to grade CD ulcer severity based on morphological features, stratifying ulcers into 3 severity levels. Cardoso et al [22] proposed a convolutional neural network–based automatic scoring system that evaluates CD severity by quantifying the percentage of positive frames in capsule endoscopy videos. Although these studies reflect disease severity based on one aspect, comprehensive assessment of CD severity remains a major challenge in AI model development as it requires not only accurate detection of ulcers and other lesions but also evaluation of lesion area and extent. The proposed temporal framework attempts to approximate this clinical process by combining lesion-level features and sequence-level distribution information.

### Limitations

Several limitations should be considered. First, lesion detection and localization and LS regression may differ in their sensitivity to shared parameters. Future work should evaluate adaptive multitask optimization strategies such as gradient normalization or dynamic loss weighting to better balance the different tasks.

Second, detection of subtle micro-ulcers remains challenging. The ulcer recall rate was 76.7%, indicating that early or small lesions may be missed. Incorporating high-resolution feature pyramids or attention-enhanced detection modules may improve sensitivity for microlesions.

Third, stenosis assessment often depends on sequential morphological changes and obstruction patterns rather than a single static frame. Although LSTM incorporated temporal information from 900 sampled frames, the central window strategy covered only approximately 7% (900/12,600, 7.1%) of a typical recording and may have missed focal lesions outside the sampled windows. In addition, stenosis classification still relied substantially on image-level features. Future work should evaluate denser or adaptive sequence sampling and full-sequence temporal models.

Fourth, the primary training and testing data were obtained from a single institution using one capsule endoscopy system. The 20-case temporal validation cohort was independent but same center and used the same device and annotation protocol; therefore, it should not be interpreted as true multicenter external validation. Prospective multicenter studies involving diverse capsule systems and independent annotators are necessary.

Fifth, specificity in control cases without CD-related inflammatory lesions was suboptimal. Of 5 control cases, 4 received low nonzero scores, suggesting that the model has not fully learned the boundary between mucosa without visible CD-related inflammatory lesions and very mild inflammatory changes. Although all scores were far below the clinical threshold for mild disease, future work should include a larger and more balanced set of control cases and non-CD populations.

Sixth, the scoring model dataset was limited to 89 cases for training and internal testing, with an additional 20 cases for same-center temporal validation. The CIs describe sampling uncertainty within these observed cohorts, but they cannot overcome the limited sample sizes, same-center design, or potential optimism of variance-based metrics in a small validation set. The temporal validation *R*^2^ and explained variance, both rounded to 1.00, should therefore be interpreted cautiously. Larger prospective multicenter cohorts with independent annotation are needed to obtain more stable uncertainty estimates and assess generalizability.

Seventh, although interobserver reliability for LS annotation was excellent, the reference standard still depended on expert judgment. Borderline lesions and ambiguous frames may introduce labeling uncertainty. Future studies should include independent expert panels from multiple centers and, where feasible, correlation with objective clinical or histopathological data.

Finally, Grad-CAM provided qualitative interpretability but did not quantify the contribution of each lesion type to the final LS. More granular explanation methods such as Shapley additive explanations, local interpretable model-agnostic explanations, attention rollout, or uncertainty-aware attribution may improve clinical transparency and facilitate physician trust.

### Future Directions

Future studies should prioritize prospective multicenter validation, inclusion of control cases and non-CD populations, calibration of confidence thresholds, and benchmarking against independent expert panels.

The model may also be evaluated as a tool for standardizing preliminary SBCE review in centers with limited expert availability, provided that final interpretation remains under physician supervision.

In addition, integration with clinical biomarkers, laboratory indicators, and other imaging modalities may support more comprehensive disease activity assessment and longitudinal monitoring.

### Conclusions

This study presents a multitask deep learning framework for automated LS estimation from SBCE in small bowel CD. Eval-Model demonstrated preliminary agreement with expert reference scores in internal testing and same-center temporal validation. These findings support further investigation of automated, sequence-level SBCE assessment, whereas prospective multicenter validation with independent external annotation remains necessary before clinical deployment.

## Abbreviations

CD: Crohn disease
CIoU: complete intersection over union
CSP: cross-stage partial
fps: frames per second
Grad-CAM: Gradient-Weighted Class Activation Mapping
LS: Lewis score
LSTM: long short-term memory
MAE: mean absolute error
mAP: mean average precision
MSE: mean squared error
RMSE: root mean squared error
SBCE: small bowel capsule endoscopy
SPPF: spatial pyramid pooling–fast
YOLO: You Only Look Once

## Data Availability

The data analyzed in this study are available from the corresponding author on reasonable request and subject to institutional data governance requirements.
